# Overcoming Immunotolerance in Chronic Hepatitis B: Efficacy of Granulocyte‐Macrophage Colony‐Stimulating Factor–Based Immunotherapy in Achieving Hepatitis B Surface Antigen Seroclearance

**DOI:** 10.1002/mco2.70676

**Published:** 2026-04-05

**Authors:** Shuang Geng, Feifei Yang, Hongyu Jia, Gan Zhao, Weidong Zhao, Jie Yu, Haoxiang Zhu, Huan Cai, Lishan Yang, Shuren Zhang, Fang Yu, Xiang Jin, Shijie Zhang, Xianzheng Wang, Yida Yang, Jiming Zhang, Bin Wang

**Affiliations:** ^1^ Key Laboratory of Medical Molecular Virology (MOE/NHC/CAMS) College of Basic Medical Sciences Fudan University Shanghai China; ^2^ Cumming School of Medicine University of Calgary Calgary Canada; ^3^ Shanghai Institute of Infectious Disease and Biosecurity Fudan University Shanghai China; ^4^ National Medical Center for Infectious Diseases Huashan Hospital Fudan University Shanghai China; ^5^ Department of Infectious Disease the First Affiliated Hospital Zhejiang University Hangzhou Zhejiang Province China; ^6^ Advaccine Biopharmaceutics (Suzhou) Co. LTD Suzhou Jiangsu Province China; ^7^ Department of Clinical Laboratory the First Affiliated Hospital of Dali University Dali Yunnan Province China; ^8^ Huapont Life Sciences Co., LTD Chongqing Chongqing China; ^9^ Genor Biopharma Co. Ltd Shanghai, Pudong New District China; ^10^ Pfizer China Beijing Beijing China; ^11^ China Overseas International Center Beijing, Xicheng District China

**Keywords:** functional cure, granulocyte‐macrophage colony‐stimulating factor, hepatitis B, hepatitis B virus surface antigen, immunotherapy, regulatory T cells

## Abstract

Chronic hepatitis B (CHB) remains incurable due to the immune system's tolerance toward the hepatitis B virus (HBV) surface antigen (HBsAg). This study aimed to achieve a functional cure by breaking HBV tolerance through immunotherapy. CHB patients were treated with either standard nucleotide analog (NA) therapy (Adefovir Dipivoxil, ADV) (Cohort 1) or ADV combined with interferon‐alpha (IFN‐α) (Cohort 2). Additionally, a third cohort received the THRIL‐GM‐Vac regimen: three low‐dose GM‐CSF injections followed by one dose of the HBV vaccine, alongside standard treatment. THRIL‐GM‐Vac treatment (Cohort 3) achieved a significant 2log10 reduction in HBsAg levels in 21.7% of participants, and 8.7% HBsAg clearance in Cohort 3 compared to 0% and 4.17% in Cohorts 1 and 2, respectively. Furthermore, THRIL‐GM‐Vac significantly reduced HBV‐specific tolerogenic T cells (Tregs), explaining the sustained HBsAg decrease. Upregulation of anti‐HBV T cell responses confirmed THRIL‐GM‐Vac's ability to disrupt HBV tolerance and enhance HBsAg‐specific cellular immunity. This suggests its potential effectiveness in treating individuals with moderate to low HBsAg levels. THRIL‐GM‐Vac treatment in Cohort 3 resulted in 8.7% HBsAg clearance alongside Treg depletion and enhanced anti‐viral T cell responses. These findings present a promising strategy to overcome immunotolerance and potentially combat chronic HBV infection.

## Graphical abstract

Chronic hepatitis B patient (CHB) presents a challenge due to immune tolerance toward HBV surface antigen (HBsAg). This study aimed to achieve a functional cure by disrupting HBV tolerance through immunotherapy. CHB received standard NA therapy, or NA combined with IFN‐α, while a third group received the THRIL‐GM‐Vac regimen. THRIL‐GM‐Vac led to a significant 2log10 reduction in HBsAg levels in 21.7% of participants, outperforming other treatments. Reducing Treg cells and enhancing anti‐HBV T cell responses indicate THRIL‐GM‐Vac's potential to combat CHB by enhancing cellular immunity and depleting Tregs. This offers a promising strategy for overcoming immunotolerance in chronic HBV infection. Created with www.Biorender.com.

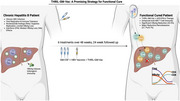

## 1 Introduction

Chronic hepatitis B (CHB) remains a formidable global health challenge, affecting over 250 million individuals worldwide and representing a leading cause of cirrhosis and hepatocellular carcinoma (HCC) [1]. Despite the availability of effective antiviral agents, the therapeutic landscape is constrained by a fundamental limitation: current nucleotide analogs (NAs) and interferon‐alpha (IFN‐α) regimens suppress viral replication but achieve functional cure—defined as durable HBsAg seroclearance—in fewer than 10% of patients after years of therapy [2, 3]. This failure stems primarily from the establishment of profound immunotolerance, driven by persistently high antigenic loads that exhaust virus‐specific CD8+ T cell responses and expand immunosuppressive regulatory T cells (Tregs) [4, 5]. Consequently, the host immune system remains anergic to HBV antigens, preventing the durable viral control necessary for functional cure and leaving patients at lifelong risk of progressive liver disease.

The clinical imperative for HBsAg seroclearance has been underscored by compelling epidemiological evidence demonstrating that individuals achieving HBsAg loss exhibit up to 60%–70% reduction in HCC incidence, even among those with established cirrhosis [6, 7]. However, spontaneous seroclearance occurs at an annual rate of merely 0.5%–2%, and NA‐induced HBsAg loss in less than 3% of patients after 5 years of continuous treatment [8]. Although pegylated IFN‐α (PEG‐IFN) regimens yield higher clearance rates of 3%–7% annually, this remains insufficient for the vast majority of CHB patients, particularly those with high baseline HBsAg levels (> 1000 IU/mL) who demonstrate particularly poor immunological responsiveness [9]. These suboptimal outcomes highlight an urgent unmet need for novel therapeutic strategies that can specifically disrupt immunotolerance and reconstitute effective anti‐HBV immunity.

Granulocyte‐macrophage colony‐stimulating factor (GM‐CSF) has emerged as a potential adjuvant capable of bridging this therapeutic gap. Originally characterized for its hematopoietic functions, GM‐CSF potently activates dendritic cells (DCs), enhances antigen processing and presentation, and promotes Th1‐polarized cellular immunity—all critical defects in CHB [10, 11, 12]. Critically, low‐dose GM‐CSF has been shown to preferentially expand myeloid DCs while avoiding excessive myeloid‐derived suppressor cell activation, suggesting dose‐dependent immunological effects that can be harnessed therapeutically [13, 14]. When combined with therapeutic HBV vaccination, GM‐CSF creates a synergistic milieu: the vaccine provides defined antigenic targets while GM‐CSF ensures optimal antigen presentation and T cell priming, potentially overcoming the anergic state that characterizes CHB immunotolerance.

While this conceptual framework was recently validated by Jia and colleagues, who demonstrated that combination therapy with PEG‐IFN‐α2b, tenofovir disoproxil fumarate (TDF), GM‐CSF, and HBV vaccine significantly improved functional cure rates in CHB patients [15, 16], a critical mechanistic question remains: can an optimized GM‐CSF and HBV vaccine regimen specifically designed to disrupt immune tolerance enhance HBsAg clearance beyond what is achievable with standard approaches? The precise dosing, timing, and immunological impact required to systematically break the tolerant state rather than simply augment immune activity remains undefined, representing a fundamental gap in translating this promising combination into a reproducible and scalable therapeutic strategy.

To directly address this question, we conducted a prospective, randomized clinical trial evaluating a novel therapeutic regimen termed THRIL‐GM‐Vac (three injections of low‐dose GM‐CSF with one dose of human HBV vaccine), strategically designed to break immunotolerance in CHB patients. Our primary aim was to assess whether this optimized, low‐dose GM‐CSF and HBV vaccine combination could enhance HBsAg seroclearance rates compared to standard IFN‐based therapy. We demonstrate that the THRIL‐GM‐Vac regimen significantly increased functional cure rates at 48 weeks, with particularly pronounced efficacy in patients with intermediate HBsAg levels (100–1000 IU/mL). Importantly, this therapeutic intensification was well‐tolerated and induced robust HBsAg‐specific T cell responses, providing a mechanistic correlate for clinical efficacy. These findings establish an optimized, clinically feasible protocol for GM‐CSF adjuvant therapy and represent a significant advance toward making functional cure an achievable goal for a broader population of CHB patients.

## 2 Results

### 2.1 Assessment of the HBsAg Seroclearance

**FIGURE 1:**
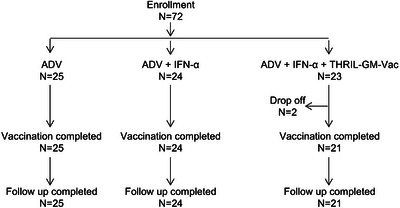
Study flowchart of patient enrollment and treatment completion. This flowchart illustrates the distribution of 72 chronic hepatitis B (CHB) patients enrolled in the study across three treatment groups and their completion status. Group 1 (ADV alone): 25 patients were enrolled, and all 25 completed the treatment and follow‐up. Group 2 (ADV + IFN‐α): 24 patients were enrolled, and all 24 completed the treatment and follow‐up. Group 3 (ADV+IFN‐α+THRIL‐GM‐Vac): 23 patients were enrolled. Two patients dropped out during the study (one due to thyroid dysfunction at Week 40 and one due to a rash at Week 24), resulting in 21 patients completing the treatment and follow‐up.

**FIGURE 2:**
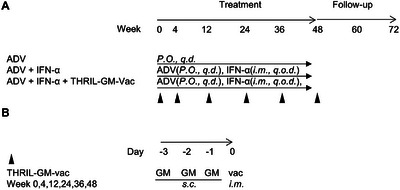
Trial design and scheme. (A) Schematic representation of the trial schedule and treatment routes for the three groups. Group 1 received oral adefovir dipivoxil (ADV) daily (*P.O., q.d*.). Group 2 received ADV plus subcutaneous injections of interferon‐alpha (IFN‐α) on alternate days (*i.m., q.o.d*.). Group 3 received ADV, IFN‐α, and the THRIL‐GM‐Vac regimen, which consisted of three consecutive daily subcutaneous injections of granulocyte‐macrophage colony‐stimulating factor (GM‐CSF) followed by an intramuscular injection of recombinant hepatitis B virus (HBV) vaccine on the fourth day. This cycle was repeated at Weeks 0, 4, 12, 24, 36, and 48, as indicated by the solid triangles. (B) Detailed schedule of GM‐CSF and HBV vaccine administration for Group 3. GM‐CSF (75 µg/dose) was administered subcutaneously for three consecutive days, followed by an intramuscular injection of HBV vaccine (20 µg/dose) on the fourth day.

**FIGURE 3:**
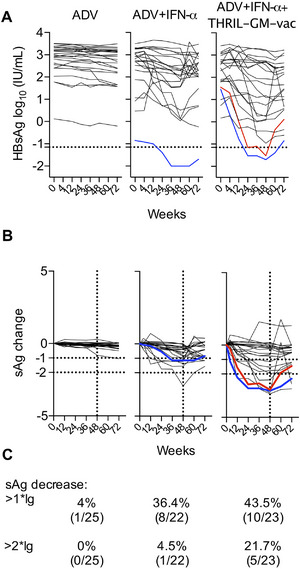
Analysis of circulating HBsAg levels before and after treatment. Serum samples were collected from each participant at baseline (Week 0) and at Weeks 12, 24, 36, 48, 60, and 72. HBsAg levels were quantified using the Roche Cobas e411 platform (Roche Diagnostics, Mannheim, Germany). (A) Dynamic changes in HBsAg levels over the treatment period. Colored lines represent individual patients who achieved HBsAg seroclearance (defined as HBsAg < 0.07 IU/mL, indicated by the dashed line). The solid line represents the mean HBsAg level for each group. (B–C) Individual normalized HBsAg dynamics. Individual HBsAg values were normalized by subtracting the baseline level for each patient. Horizontal dashed lines represent 1‐log and 2‐log decreases in HBsAg. Vertical dashed lines indicate the end of treatment at Week 48. Solid lines represent the HBsAg dynamics for each patient. Statistical analysis was performed using the Mann–Whitney *U* test for comparisons between groups at each time point.

A total of 72 CHB patients were enrolled in the study and randomly allocated into three treatment groups: Group 1 (ADV‐only, 25 patients), Group 2 (ADV+IFN‐α, 24 patients), and Group 3 (ADV+IFN‐α+THRIL‐GM‐Vac, 23 patients) (Figure 1). Treatment scheme was detailed in Section 4 (Figure 2). The baseline characteristics, including age, gender, HLA‐A2 expression, ALT, AST, HBsAg, and white blood cell count, are presented in Table 1, and there were no significant differences. Two patients in Group 3 discontinued the trial due to adverse events: one experienced thyroid dysfunction at Week 40, and the other developed a rash at Week 24. HBsAg seroclearance, defined as the complete clearance of serum HBsAg, was monitored as the primary endpoint of the study. Patients in Group 1 (ADV‐only) showed no seroclearance throughout the trial, and HBsAg levels generally remained unchanged. In Group 2 (ADV+IFN‐α), one patient (4.17%) achieved seroclearance at Week 24, but only after starting with a low baseline HBsAg level. In contrast, two patients in Group 3 (ADV+IFN‐α+THRIL‐GM‐Vac, 8.7%) achieved seroclearance at Week 24 and Week 36, respectively (Figure 3A), and maintained a negative HBsAg status at the 24‐week follow‐up post‐treatment discontinuation.

**TABLE 1:** The baseline characteristics of the patients.

Baseline characteristics	ADV (*n* = 25)	ADV+IFNα (*n* = 24)	ADV+IFNα+THRIL‐GM‐vac (*n* = 23)	Total (*n* = 72)	*p* value
Males’ *n* (%)	17 (68.0)	22 (95.8)	20 (87.0)	60 (83.3)	0.0659
Age (years)	44.0 (39.0–55.0)	40.0 (32.0–47.5)	39.0 (30.0–50.0)	42.0 (33.0–52.0)	0.111
HBsAg (log_10_ IU/mL)	2.99 (2.48–3.27)	2.79 (1.99–3.17)	2.64 (1.78–2.97)	2.78 (2.11–3.19)	0.0647
ALT (IU/L)	22.0 (18.0–29.0)	28.0 (21.0–41.0)	26.0 (19.0–46.0)	26.0 (20.0–35.0)	0.180
AST (IU/L)	22.0 (18.0–28.0)	20.0 (18.5–24.0)	22.0 (19.0–25.0)	21.0 (19.0–25.0)	0.884
WBC (*10^9^/L)	5.3 (4.5–5.8)	5.5 (4.9–6.7)	5.3 (4.7–6.6)	5.3 (4.8–6.4)	0.628
HLA‐A2, positive (%)	14 (56.0)	12 (50.00)	12 (52.2)	38 (52.8)	0.913

*Note*: Values are expressed as median (IQR) or the number of patients (%).

Abbreviations: ALT, alanine‐aminotransferase; AST, aspartate‐aminotransferase; HBsAg, Hepatitis B surface antigen; HLA, human leukocyte antigens; WBC, white blood cell.

### 2.2 Treatment Efficacy and Immune Mechanism

Treatment efficacy was further evaluated based on HBsAg reduction from baseline (Week 0). Patients achieving reductions of > 1‐log10 and > 2‐log10 were classified as treatment responders. Group 1 showed minimal effects, with only one patient exhibiting a 1‐log10 reduction and none achieving a 2‐log10 decrease. In Group 2, 33.33% (8/24) achieved a > 1‐log10 decrease, and 4.17% (1/24) achieved a > 2‐log10 decrease. Group 3 demonstrated the highest efficacy: 43.5% (10/23) achieved a > 1‐log10 decrease, and 21.7% (5/23) achieved a > 2‐log10 decrease, including the two patients with seroclearance (Figure 3B,C).

**FIGURE 4:**
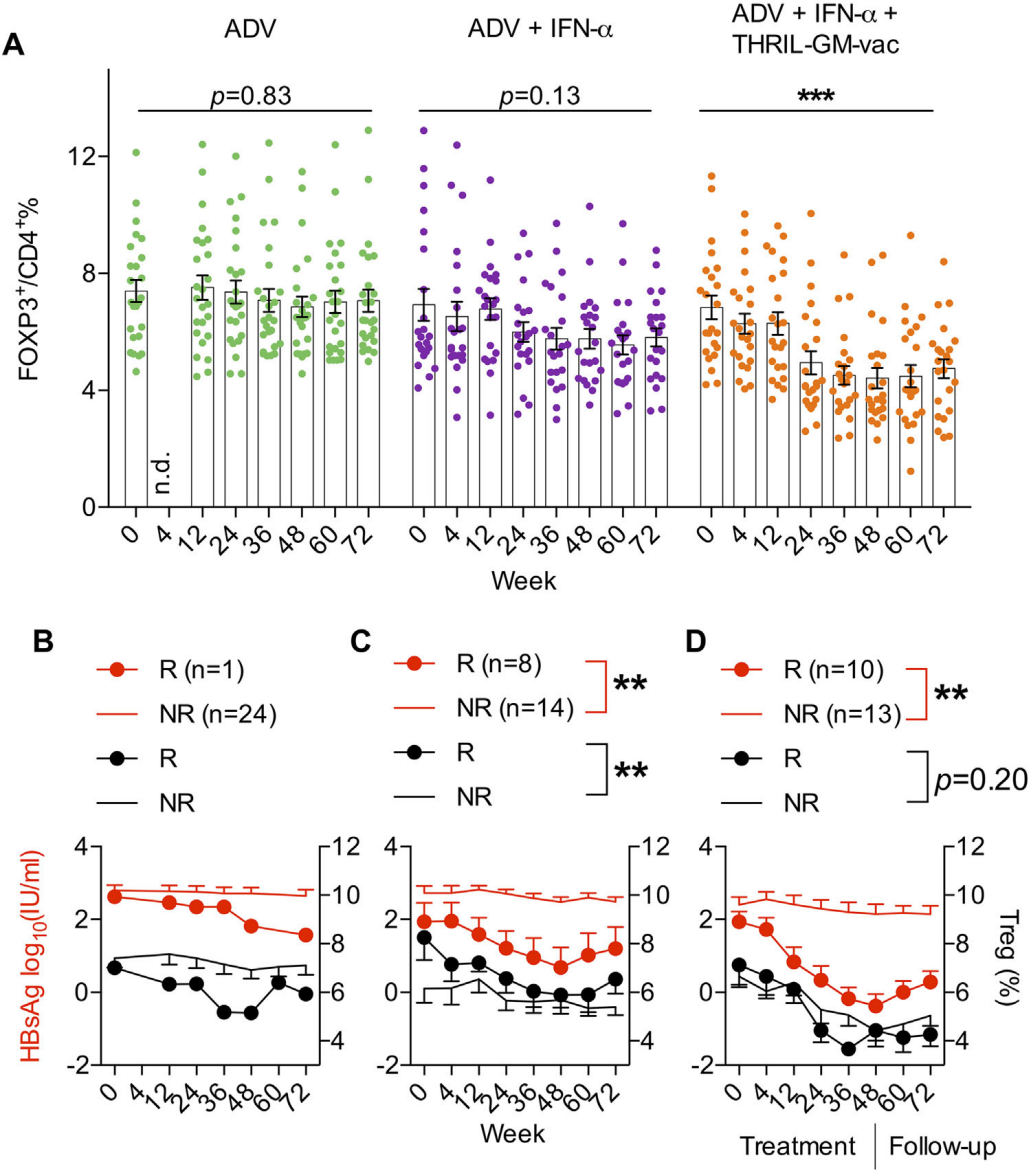
Treg cell dynamic changes throughout treatment. Peripheral blood mononuclear cells (PBMCs) were isolated from all patients at baseline and at Weeks 12, 24, 36, 48, 60, and 72. PBMCs were stimulated in vitro with HBsAg peptide pools (10 µg/mL) for 8 h in the presence of anti‐CD28 (0.1 µg/mL) and brefeldin A (BD Biosciences). (A) Changes in HBsAg‐specific CD4 + FoxP3 + Treg cells. This panel shows the percentage changes in HBsAg‐specific CD4 + FoxP3 + Treg cells in patients' PBMCs throughout treatment. Each dot represents an individual patient. Statistical significance was determined using the Wilcoxon signed‐rank test for paired comparisons (***p < 0.001, Week 72 vs. Week 0). (B–D) Correlation between HBsAg levels and Treg percentages in Group 1 (B), Group 2 (C), and Group 3 (D). The red line represents the HBsAg level, the black line represents the Treg percentage, and the dotted line represents responders (R). Plain lines represent non‐responders (NR). Pearman's coefficient correlation analysis was used to assess the correlation between HBsAg levels and Treg percentages (**p < 0.01).

As Treg are a significant tolerance determinant for chronic HBV infection, HBsAg‐specific CD4+FoxP3+ Treg cells were analyzed by flow cytometry from the patient's peripheral blood mononuclear cells (PBMC) and evaluated both during the therapy and during the follow‐up period (Figure S1). The HBsAg‐specific Treg cells were not significantly affected by the ADV‐only therapy (Group 1), however, the numbers of Treg cells did decrease under the ADV+IFN‐α combination treatment (Group 2), albeit not to a statistically significant change. Treg cells that were antigen‐specific dropped by a substantial amount in Group 3, which received therapy with THRIL‐GM‐Vac (Figure 4A, Figure S1B).

### 2.3 Correlation Between HBsAg and Treg Levels

To better understand the correlation between HBsAg reduction and changes in Foxp3+Treg, responders and non‐responders were compared. Figure 4B–D illustrates the association between the subjects' levels of HBsAg and the changes in antigen‐specific Treg levels that occurred over time in Group 1 (Figure 4B), Group 2 (Figure 4C), and Group 3 (Figure 4D). After receiving therapy, “responders” had lower levels of HBsAg, which is linked with lower levels of Treg cells. The results show a significant negative correlation between HBsAg levels and Treg percentages in Group 3, indicating that the reduction in HBsAg is associated with a decrease in Treg cells. Pearman's coefficient correlation analysis was used to assess the correlation (**p < 0.01). This suggests that the loss of HBsAg may be a result of breaking immunological tolerance. When compared to responders, non‐responders showed much lower declines in Tregs, which is consistent with the notion that the reduction in Tregs is coupled with the decrease in HBsAg.

### 2.4 Teff Cells Negatively Correlated With HBsAg (sAg) in Responders

**FIGURE 5:**
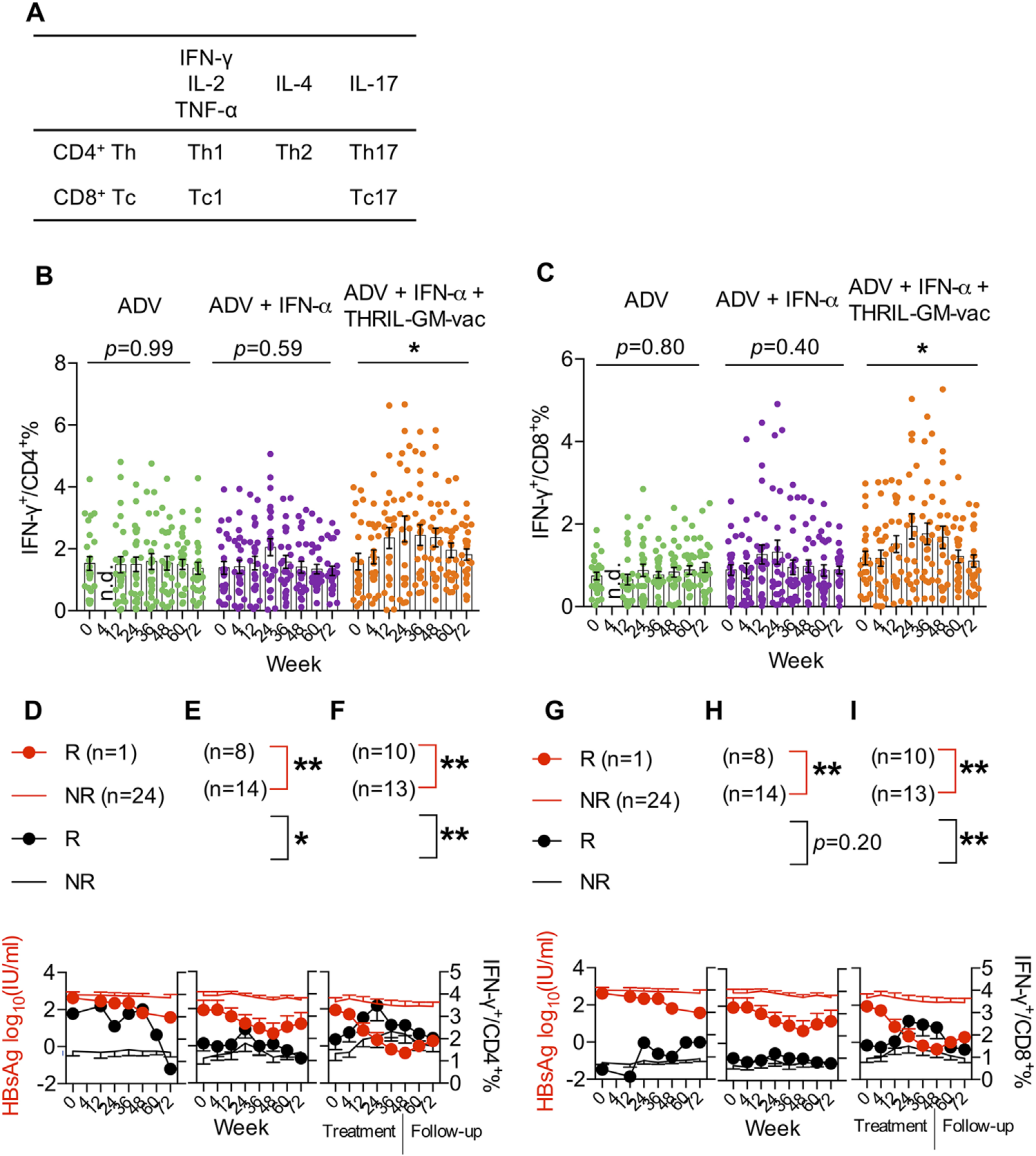
Correlation of helper CD4 T cells and cytotoxic CD8 T cells in HBsAg seroclearance. Flow cytometry analysis was performed on PBMCs gated on HBsAg peptide‐stimulated CD4+ and CD8+ T cells with intracellular staining for IL‐2, TNF‐α, IL‐17, IL‐4, or IFN‐γ. (A) Cytokines tested and indicated T cell subsets. (B) Percentage of Th1 (IFN‐γ+ in CD4+ T cells) in total CD4+ T cells. (C) Percentage of Tc1 (IFN‐γ+ in CD8+ T cells) in total CD8+ T cells. Dots represent individual patients. (D–F) Th1 negatively correlated with HBsAg levels in responders. (G–I) Tc1 negatively correlated with HBsAg levels in responders. Pearman's coefficient correlation analysis was used to assess the correlation between Th1/Tc1 percentages and HBsAg levels (*p < 0.05; **p < 0.01).

As HBV tolerance suppresses antiviral immunity, including a reduction in the frequencies of CD4+ helper T cells and CD8+ cytotoxic T cells [17], we next investigated whether the breakdown of HBV tolerance and the related decrease in Treg were reciprocally paralleled by increasing levels of HBsAg epitope‐specific CD4+ and CD8+ Teff cell activation. The effector‐cell populations were characterized by flow cytometry analysis performed on PBMCs gated on HBsAg peptide‐stimulated CD4+ and CD8+ T cells with intracellular staining for IL‐2, TNF‐α, IL‐17, IL‐4, or IFN‐γ according to Figure 5A, Figures S2A and S3A. The notion that immunological activation occurred after the tolerance break is supported by the fact that the IFN‐γ response was considerably activated in Group 3, in both Th1 cells (IFN‐γ+ in CD4+ T cells) in total CD4+ T cells (Figure 5B, Figure S2B) and Tc1 cells (IFN‐γ+ in CD8+ T cells) in total CD8+ T cells (Figure 5C, Figure S3B). In Figure 5D–I, the data on IFN‐γ and antigen from responders and non‐responders are plotted side by side to illustrate correlations between HBsAg decrease and anti‐viral cytokine dynamics. This patient's anti‐viral immune responses, rather than the anti‐viral drug action, may have affected viral replication during the ADV treatments; perhaps the patient was a spontaneous responder rather than a drug responder. There was only one responder in the ADV‐treatment group (Group 1), and this patient had more robust Th1 and Tc1 responses than the other patients in the group (Figure 5D,G). The responders in Group 2 had more robust Th1 and Tc1 responses (Figure 5E,H), but antigen clearance was not substantially different when compared with the non‐responders. Responders in Group 3 had upregulation of Th1 even in the early period (Week 4 to Week 24), followed by sequential activation of Tc1 from Week 12 through the end of treatment at Week 48 (Figure 5F,I). These findings revealed significant negative correlations with HBsAg (sAg) in responders who had the robust Th1 and Tc1 responses. During this time, the levels of HBsAg decreased the most noticeably from Week 4 to the completion of the therapy.

### 2.5 ALT Dynamics Over the Treatment Period in Each Patient

**FIGURE 6:**
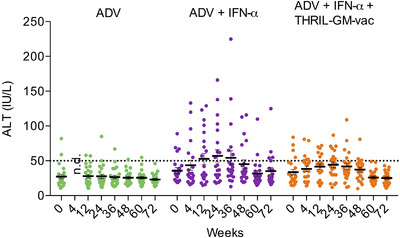
ALT dynamics over the treatment period in each patient. Serum alanine aminotransferase (ALT) levels were measured using a fully automatic biochemical analyzer (Hitachi 7600, Tokyo, Japan). The dashed line represents the normal ALT level (40 IU/L). Each dot represents an individual patient. Statistical analysis was performed using the Kruskal–Wallis test for comparisons among groups. There were no statistically significant differences among the groups (*p* > 0.05).

Liver damage is the key safety issue for ideal CHB immunotherapeutic treatment; therefore, we additionally examined any dynamic variations in ALT levels that occurred in each patient over the course of the trial. As can be seen in Figure 6, ADV therapies had no effect on ALT levels, which reflects their inability to activate the immune system. After therapy, the ALT levels of several patients in the ADV+IFN group (Group 2) increased to high but acceptable levels, suggesting that they had minor liver injury. It seems that immunotherapy with THRIL‐GM‐Vac is responsible for raising the ALT levels in Group 3, as shown by an almost twofold increase in average ALT levels at Week 24, followed by a subsequent reduction in those levels. This may reflect a positive benefit caused by the targeted clearance of infected HBV hepatocytes by antigen‐specific immune destruction.

### 2.6 Association Between Baseline HBsAg Levels and Treatment Outcomes

**FIGURE 7:**
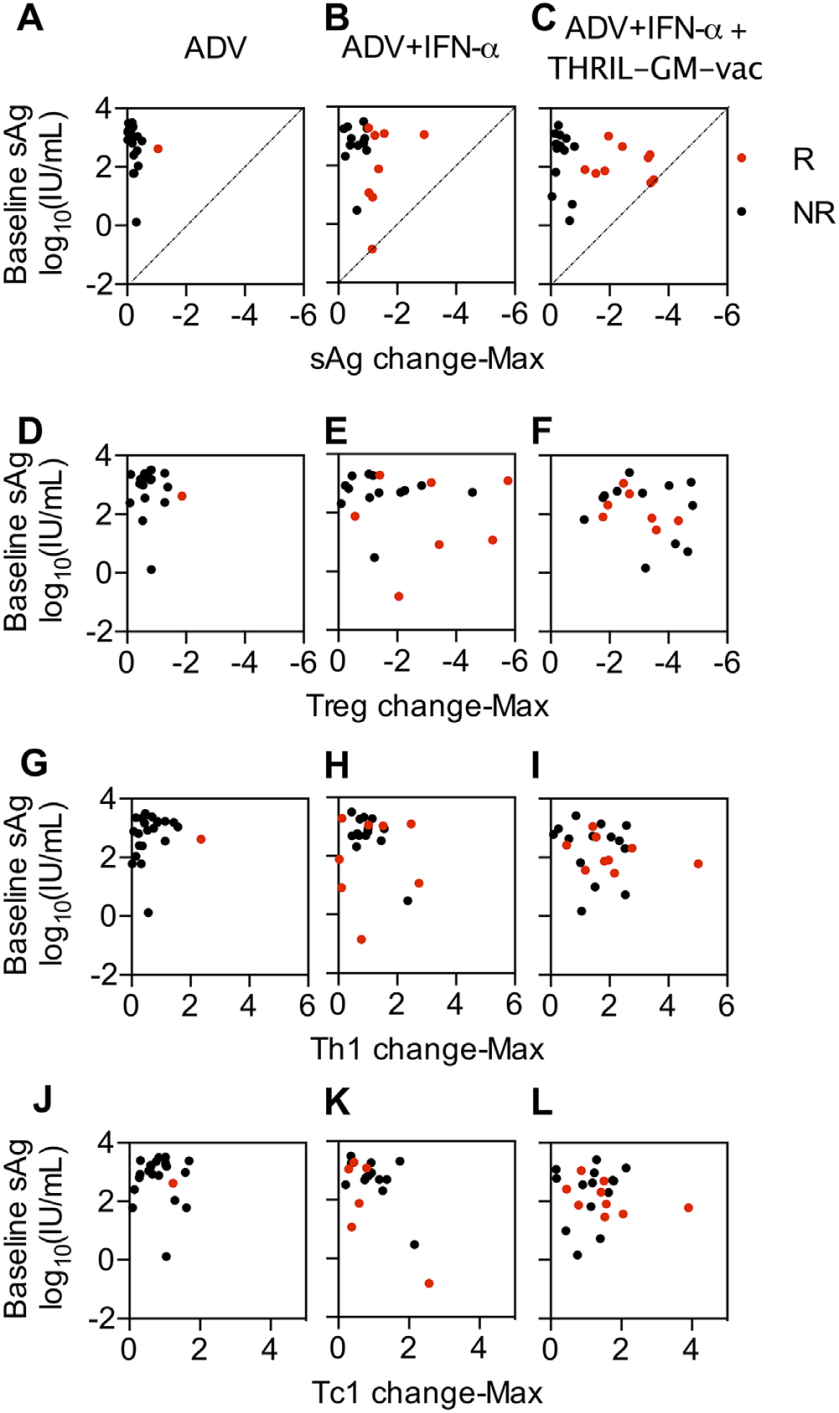
Relationship of each patient's HBsAg baseline with HBsAg reductions and immune profile changes during treatment. (A–C) Relationship between baseline HBsAg levels and HBsAg reductions at Week 48. The non‐responders (NR) are indicated as black dots, and the responders (*R*) as red dots. (D–L) Relationship between baseline HBsAg levels and changes in Treg (D–F), Th1 (G–I), and Th2 (J–L) during treatment. Pearman's coefficient correlation analysis was used to assess the correlations (**p < 0.01).

Our clinical experiences have led us to believe that the amount of HBsAg present at the beginning of therapy may determine its effectiveness. For instance, individuals who had already achieved low HBsAg baseline levels due to NA therapies did not benefit significantly from IFN‐α therapy. This observation was also evident in patients of Group 2 who received ADV+IFN‐α treatment in this study. Therefore, we analyzed the association between baseline HBsAg levels and the maximum changes in HBsAg, Treg, Th1, and Th2 during the treatment period using the Pearson correlation coefficient (Figure 7). The non‐responders tended to cluster in the upper‐left corner of each panel, indicating that higher baseline antigen levels were associated with lower reductions in HBsAg (Figure 7A–C). Responders were distributed near the diagonal HBsAg clearance line (HBsAg < 0.07 IU/mL). In Group 2 (ADV+IFN‐α), only one patient achieved seroclearance, and this patient had a relatively low baseline HBsAg level of 0.14 IU/mL. Patients with higher baseline levels exhibited only modest reductions in HBsAg. However, in Group 3 (ADV+IFN‐α+THRIL‐GM‐Vac), two patients with medium‐to‐low baseline HBsAg levels (36.36 and 29.32 IU/mL) achieved seroclearance and HBsAb production, with levels of 145 and 635 mIU/mL at 48 weeks, respectively. Additionally, patients with higher baseline levels in Group 3 showed greater reductions in HBsAg toward the clearance line (Figure 7C).

### 2.7 Immune Profile Changes

Circulating HBsAg elevation is often associated with more severe immunological exhaustion and impaired antiviral immune responses. Effective therapy has been shown to reduce Treg cells and upregulate Th1 and Tc1 responses, particularly in patients with medium‐to‐low baseline HBsAg levels (10–1000 IU/mL) [18]. We plotted the changes in Treg and Th1 cells against the baseline HBsAg levels to determine whether THRIL‐GM‐Vac could reverse immune impairment even in patients with higher baseline antigen levels. When compared to ADV monotherapy, the addition of IFN‐α to ADV (Group 2) resulted in a modest increase in the percentage of patients with substantial immune response alterations (Figure 7D,E,G,H,J,K). However, when THRIL‐GM‐Vac was added to ADV+IFN‐α (Group 3), the majority of patients demonstrated significant improvements in cellular immune responses, regardless of the initial antigen load (Figure 7D,F,G,I,J,L). This suggests that THRIL‐GM‐Vac is effective in enhancing immune activation and overcoming immunotolerance, even in patients with higher baseline HBsAg levels.

## 3 Discussion

In pursuit of an HBV functional cure, we present our first clinical effectiveness results of a new GM‐CSF‐based intervention in chronic HBV patients (CHB). Cohort 3 treated with THRIL‐GM‐Vac therapies achieved significant HBsAg reductions (2log10) in 21.7% of participants, compared to 0% in Cohort 1 and 4.17% in Cohort 2. This three‐ to fivefold improvement over standard‐of‐care demonstrates that multiple‐low‐doses of GM‐CSF, when strategically administered as an adjuvant to HBV vaccination, can quantitatively reverse the anergic state that characterizes CHB. For example, Patient 3–017, a 38‐year‐old male with baseline HBsAg of 1847 IU/mL, demonstrated progressive HBsAg decline from Week 4 onward, achieving seroclearance at Week 24 and maintaining HBsAb titers of 635 mIU/mL through Week 72. This case contrasts sharply with matched controls in Cohorts 1 and 2, where patients with comparable baseline antigenemia showed < 0.5 log10 reduction over the entire treatment period. The mechanistic correlate was equally compelling: Patient 3–017 exhibited a 68% reduction in HBsAg‐specific Tregs by Week 12, concurrent with a 4.2‐fold expansion of IFN‐γ+ CD8+ Tc1 cells, illustrating the direct link between immune reconstitution and virological outcome.

First, our data reveal that the THRIL‐GM‐Vac regimen disrupts HBsAg‐driven immunotolerance through coordinated effects on both regulatory and effector compartments. The 8.7% seroclearance rate in Group 3, while modest in absolute terms, represents a 100% relative improvement over IFN‐α monotherapy and an infinite improvement over NA alone. Critically, responders displayed a distinct immunological trajectory: HBsAg‐specific Tregs declined precipitously within the first 12 weeks (median reduction 61.3%, IQR 48.2%–74.1%), preceding the maximal decline in HBsAg levels that occurred between Weeks 24–48. This temporal dissociation suggests that Treg depletion may be a prerequisite rather than a consequence of antigen clearance. In one non‐responder case with baseline HBsAg of 4223 IU/mL, Treg suppression was transient (only 22% reduction at Week 12) and unsustained, coinciding with failure to activate HBsAg‐specific CD8+ T cells above baseline. Conversely, another responder who achieved a 2.8 log10 reduction without full seroclearance maintained Treg suppression > 50% through Week 48 while mounting vigorous Th1 responses, suggesting that durable Treg control is necessary but not sufficient for complete antigen loss. These case‐specific analyses underscore that the quality and sustainability of immune modulation, rather than its mere initiation, determine therapeutic success.

Second, the post‐treatment rebound in HBsAg observed in some responders raises important questions about the durability of immune‐mediated control. While Tregs remained suppressed at Week 72, declining effector T cell frequencies (both Th1 and Tc1) correlated with antigen resurgence, suggesting that memory T cell establishment was incomplete. For instance, one responder who achieved 2.1 log10 HBsAg reduction by Week 48 with undetectable Tregs saw HBsAg rebound to within 0.5 log10 of baseline by Week 60 as HBsAg‐specific IFN‐γ+ CD8+ cells contracted from 4.8% to 1.2% of total CD8+ T cells. This pattern mirrors observations in acute HBV resolution, where long‐term control requires establishment of resident memory T cells in the liver—a process that may require > 48 weeks of antigenic stimulation. The transient nature of vaccine‐induced immunity, administered only six times over one year, likely explains this attenuation. We hypothesize that extending THRIL‐GM‐Vac administration to 72–96 weeks, or incorporating quarterly booster vaccinations, could consolidate effector‐to‐memory transition. Alternatively, integrating immune checkpoint inhibitors (e.g., anti‐PD‐1) during Weeks 36–48 might prevent Teff exhaustion and preserve functional cure.

Third, our results demonstrate that patient stratification by baseline immune profile, rather than solely by virological parameters, may optimize therapeutic outcomes. While baseline HBsAg levels proved predictive of response (median 1247 IU/mL in responders vs. 2863 IU/mL in non‐responders, *p* = 0.008), immunological heterogeneity was equally decisive. One non‐responder case, despite a relatively low baseline HBsAg of 892 IU/mL, failed to respond due to an exceptionally exhausted T cell phenotype. This case exemplifies why composite biomarkers combining antigen load (HBsAg < 1000 IU/mL) and immune fitness are needed. Our ongoing analyses of myeloid‐derived suppressor cell (MDSC) frequencies at baseline [19] indicate that cases with an MDSC frequency in the range of PBMCs (< 1.99%) exhibited superior HBsAg seroclearance, suggesting that multi‐parameter immune staging could identify the ∼30% of patients most likely to benefit from THRIL‐GM‐Vac, sparing others unnecessary immunotherapy.

Fourth, our findings corroborate and extend the classical paradigm that sequential Th1 and Tc1 activation is required for durable HBsAg clearance, but with important dose‐schedule nuances. The THRIL‐GM‐Vac protocol triggered early Th1 polarization (IFN‐γ+ CD4+ cells peaking at Week 12) followed by Tc1 expansion (IFN‐γ+ CD8+ cells peaking at Week 24), temporally aligning with the decline in HBsAg. This kinetics mirrors spontaneous HBV resolution but is pharmacologically accelerated. Notably, one seroconverter displayed a rapid Tc1 response by Week 12 (3.1% of CD8+ cells) that was sustained through Week 48 (5.4%), whereas a partial responder showed delayed Tc1 activation (Week 24) that waned prematurely (Week 36). These differential kinetics suggest that adjuvant timing could be personalized: patients with robust baseline HBV‐specific CD8+ precursors might benefit from earlier vaccine boosting (Week 8 instead of 12) to capitalize on their proliferative capacity, while those with depleted pools may require extended GM‐CSF priming to expand DCs before antigen exposure. Such schedule optimization, guided by real‐time immune monitoring, could potentially increase seroclearance rates from 8.7% to the 15%–20% range seen in optimized PEG‐IFN studies.

While our GM‐CSF‐based strategy demonstrated significant improvement over frontline NA or IFN‐α treatments, we recognized several limitations. The translational gap between near‐complete efficacy in HBV transgenic mice and the 8.7% seroclearance in humans reflects fundamental differences in immune tolerance ontogeny: mouse models typically involve perinatal antigen exposure over weeks, whereas human CHB represents decades of tolerogenic conditioning. One case with the highest baseline antigenemia in our cohort (8432 IU/mL) exemplified this limitation—despite full regimen compliance and marked Treg depletion (61% reduction), antigen‐specific CD8+ T cells remained functionally impaired, producing only IL‐10 but not IFN‐γ upon stimulation, indicative of terminally exhausted cells that resist reprogramming. This highlights that THRIL‐GM‐Vac is most effective in patients with “plastic” immune tolerance (moderate antigen load, partially preserved T cell stemness) rather than irreversible exhaustion.

In conclusion, the THRIL‐GM‐Vac regimen represents a paradigm shift in CHB therapy by demonstrating that strategically timed, low‐dose GM‐CSF combined with HBV vaccination can reproducibly break immunotolerance and achieve functional cure rates substantially higher than conventional IFN‐based therapy. Our findings establish a clear clinical and immunological rationale for integrating optimized adjuvant immunotherapy into the CHB treatment algorithm, particularly for patients with intermediate HBsAg levels who represent a large yet underserved population. While these results warrant validation in larger multicenter studies with extended post‐treatment follow‐up to confirm durability, they provide an immediately implementable protocol that could transform clinical practice. Future investigations should explore predictive biomarkers of response, optimal retreatment strategies for non‐responders, and applicability to diverse patient subgroups, including those with cirrhosis or low HBsAg titers. Ultimately, this work advances the field beyond simple viral suppression toward engineered immune modulation, bringing us closer to making a functional cure an achievable reality for millions of CHB patients worldwide.

## 4 Materials and Methods

### 4.1 Drugs

Commercial products, Adefovir Dipivoxil Tablets at 10 mg/tablet were manufactured under GMP‐compliant conditions and donated by Cisen Pharmaceutical Co., Ltd (Jinan, Shandong, China), Recombinant Human Interferon‐α2b Injection at 5 mIU/injection by Beijing Kawin Technology, and human recombinant GM‐CSF (Topleucon) at 75 µL/dose were manufacturaly produced under GMP‐compliant conditions in E. Coli and donated by Amoytop Biotech (Xiamen, Fujiang, China). Human recombinant HBV vaccines 20 µg/dose were manufactured and produced under GMP‐compliant conditions in the yeast system and donated to the study by Kantai Biologicals (Shenzhen, China). The vaccines are based on the prevalent strain of HBV in China (ADR).

### 4.2 Patient Enrollment and Treatment Schedules

A total of 72 CHB patients were enrolled from Huashan Hospital between September 2013 and March 2014 and randomly allocated into three treatment groups: Group 1 (ADV‐only, 25 patients), Group 2 (ADV+IFN‐α, 24 patients), and Group 3 (ADV+IFN‐α+THRIL‐GM‐Vac, 23 patients) (Figure 1). Eligible enrollments were determined as: they were aged between 18 and 65 years, had previously received NA therapy, were HBeAg‐negative, had HBV DNA below the detection limit, HBsAg levels < 5000 IU/mL, and had no contraindications to interferon treatment. Key baseline characteristics, including HLA‐A2 expression and prior treatment history, were recorded to assess their impact on treatment outcomes. Patients were excluded from the study if they met the following criteria: They were co‐infected with hepatitis C, D, or E virus or HIV; had any serious illnesses that might confound their outcomes, including any uncontrolled, clinically significant renal, cardiac, pulmonary, vascular, neurogenic, digestive, and metabolic disorders (such as thyroid disorders or adrenal disease) or cancer; had decompensated liver cirrhosis or liver failure; and had any other known disease for which interferon therapy was not suitable.

ADV was administered orally every day (*q.d*.), IFN‐α was administered as an intramuscular (*i.m*.) injection on alternate days (*q.o.d*.), and THRIL‐GM‐Vac was administered as daily (*q.d*.) subcutaneous (*s.c*.) injections of GM‐CSF for three consecutive days followed by an *i.m*. injection with Human HBV Vaccine at the same site on the fourth day of Weeks 0, 4, 12, 24, 36, and 48 (Figure 2).

### 4.3 Biochemical and Serological HBV Analysis

Serum alanine aminotransferase activity (ALT) was determined by a fully automatic biochemical analyzer (Hitachi 7600, Tokyo, Japan). Serum HBsAg, HBsAb, and HBeAg were measured using enzyme immunoassay equipment (Cobas e411, Roche Diagnostics, Mannheim, Germany).

### 4.4 Virological Studies

Serum HBV DNA, HBeAg seroconversion, and the levels of serum HBsAg and ALT were monitored by an independent third‐party laboratory (Clinical Laboratory, Ruijin Hospital, Shanghai, China), using the same lots of reagents. Sequential samples from one patient were tested on the same day. Abbott EIA AxSYM (Abbott, Abbott Park, IL, USA) was employed for the detection of HBsAg, HBeAg, and anti‐HBe. HBV DNA was quantified by fluorescent PCR assay (PiJi, Shenzhen Co, China, with a detection limit of 500 copies/mL). Architect HBsAg QT assay (Abbott, Abbott Park, IL, USA) was used for serum HBsAg quantification.

### 4.5 Immunological Assays

PBMCs were isolated from fresh blood by Lymphoprep (Axis‐Shield, Norway) and re‐suspended in complete medium R10 (RPMI 1640 supplemented with 25 mM HEPES, 2 mM L‐glutamine, 100 U/mL penicillin, 100 µg/mL of streptomycin, and 10% fetal calf serum, all from Gibco (Life Technology, USA). For T cell expansion in vitro, PBMC were suspended in R10 in the presence of anti‐CD3 (0.1 µg/mL; Miltenyi Biotec, USA) and anti‐CD28 (0.05 µg/mL, Miltenyi Biotec) at a concentration of 5 × 106 cells/mL and seeded at 1 mL/well in a 12‐well plate. The immunological assays were performed on Day 3 of the expansion. Antigen‐specific stimulation, expanded PBMC were washed once with R10 and then stimulated with HBV peptide pools (10 µg/mL, listed in Table S1) or medium alone (control) for 8 h in the presence of anti‐CD28 (0.1 µg/mL; Miltenyi Biotec) and brefeldin A (BD Biosciences). Cells incubated with anti‐CD3 (1 µg/mL) and anti‐CD28 (100 ng/mL) were used as positive controls. The plates were incubated at 37°C in a humidified atmosphere with 5% CO_2_ for 72 h before the cells were labeled with specific monoclonal antibodies and subjected to flow cytometry analysis.

### 4.6 Flow Cytometry Detection

Cells after washing were stained with anti‐CD4 and anti‐CD8 monoclonal antibodies for 30 min at 4°C, then fixed and permeabilized using 4% paraformaldehyde (PFA, Sinopharm Chemical Reagent Co., Ltd, China) and 0.2% Triton X‐100 (Genview, China). For intracellular cytokine staining, the cells were further stained with the selected fluorescent‐labeled anti‐human monoclonal antibodies used for flow cytometric analysis: CD8 (SK1), CD4 (OKT4), FoxP3 (PCH101), IFN‐γ (4S.B3), and IL‐2 (MQ1‐17H12) from eBioscience, TNF‐α (Mab11), IL‐4 (MP4‐25D2), and IL‐17 (BL168) from BioLegend. In cytokine detection, PMA (100 ng/mL) and ionomycin (1 mg/mL) were used as positive control stimulants. An LSRFortessa (BD Biosciences) was used for flow cytometry analyses.

### 4.7 Statistics

Categorical variables were described using percentages. Parametric continuous variables were presented as means ± standard error of mean (SEM), while non‐parametric continuous variables were expressed as the median (IQR). For continuous variables, the Mann–Whitney *U* test was used for abnormal distribution, the two‐tailed Student's *t*‐test for normal distribution in two‐group comparisons, and one‐way ANOVA for three‐group comparisons. A *p* value of < 0.05 was deemed significant for all analyses.

## Author Contributions

B.W., J.Z., H.J., and Y.Y. designed the clinical study and analyzed the results. J.Z., H.J., and Y.Y. managed the operation of trials. F.Y., H.C., and L.Y. managed patient screenings, samplings, recordings, and clinical analysis. S.G., G.Z., J.Y., W.Z., S.Z., F.Y., X.J., H.Z., W.X., and S.Z. performed CMI experiments and analyzed data. S.G., B.W., and J.Z. wrote and edited the manuscript. All authors have read and approved the final manuscript.

## Ethics Statement

This clinical trial was registered in the Chinese Clinical Trial Registry (ChiCTR‐TRC‐13003254) as a multicenter and randomized study. This study was approved by the Research Ethics Committee of Huashan Hospital, Fudan University (N.2013‐294). Here, we used frozen PBMCs of three cohorts of patients from one of ten participating hospitals. Each participant in the study was enrolled in Huashan Hospital, Fudan University, after being given an informed written consent form and signed it.

## Conflicts of Interest

Author Gan Zhao was employed by Fudan University during the study period and is currently an employee at Advaccine Biopharmaceutics Co. He has no potential relevant financial or non‐financial interests to disclose. Author Shuren Zhang was a graduate student at Fudan University during the study period and is now an employee at Huapont Life Science Co. He has no potential relevant financial or non‐financial interests to disclose. Author Fang Yu was a graduate student at Fudan University during the study period and is now an employee at Genor Biopharma Co. She has no potential relevant financial or non‐financial interests to disclose. Author Xiang Jin was a graduate student at Fudan University during the study period and is now an employee at Pfizer Co. He has no potential relevant financial or non‐financial interests to disclose. Author Shijie Zhang was a graduate student at Fudan University during the study period and is now an employee at Advaccine Biopharmaceutics Co. She has no potential relevant financial or non‐financial interests to disclose. Author Bin Wang is a professor at Fudan University during the study period and has been a founder of Advaccine Biopharmaceutics Co. He has a potential relevant financial interest to disclose. The other authors declare no conflicts of interest.

## Supporting information



Supporting file 1: mco270676‐sup‐0001‐SuppMat.pdf

## Data Availability

The data that support the findings of this study are available from the corresponding author upon request. There are no restrictions on data availability.
