# Supplementary material for: Overcoming Immunotolerance in Chronic Hepatitis B: Efficacy of Granulocyte‐Macrophage Colony‐Stimulating Factor–Based Immunotherapy in Achieving Hepatitis B Surface Antigen Seroclearance
Source: MedComm (2020). 2026 Apr 5;7(4):e70676. doi: 10.1002/mco2.70676 (PMC13051871; doi:10.1002/mco2.70676)
Supplement: Supplementary file 1 — Supporting file 1: mco270676‐sup‐0001‐SuppMat.pdf [file MCO2-7-e70676-s001.pdf]

SUPPLEMENTARY INFORMATION

Overcoming Immunotolerance in Chronic Hepatitis B: Efficacy of Granulocyte Macrophage Colony Stimulating Factor Based Immunotherapy in Achieving Hepatitis B Surface Antigen Seroclearance

Shuang Geng[1,a,#], Feifei Yang[2,3,#], Hongyu Jia[4,#], Gan Zhao[1,b], Weidong, Zhao[1,c], Jie Yu[3], Haoxiang Zhu[3], Huan Cai[4], Lishan Yang[4], Shuren Zhang[1,d], Fang Yu[1, e], Xiang Jin[1,f], Shijie Zhang[1,g] Xianzheng Wang[1,h], Yida Yang[4,*], Jiming Zhang[1,2,3,*], and Bin Wang[1,2*]

[1]Key Laboratory of Medical Molecular Virology (MOE/NHC/CAMS), [2]Shanghai Institute of Infectious Disease and Biosecurity, and [3]National Medical Center for Infectious Diseases, Huashan Hospital, Fudan University, Shanghai, China.

[4]Department of Infectious Disease, The First Affiliated Hospital, Zhejiang University.

[#, *]All authors contributed equally.

To Address Corresponding Authors: Dr. Bin Wang, School of Basic Medical Sciences, Fudan University, 131 Dong An Road, 409 Fuxing Building, Shanghai, China 200032. E-mail: bwang3@fudan.edu.cn; Dr. JiMing Zhang, National Medical Center for Infectious Diseases, Huashan Hospital, Fudan University, Shanghai, China; Email:jmzhang@fudan.edu.cn; or Dr. Yida Yang, Department of Infectious Disease, The First Affiliated Hospital, Zhejiang University, Hangzhou City, Zhejiang Province, China. yidayang65@zju.edu.cn.

Supplementary Figures 1-3 and Supplementary Table.

SUPPLEMENTARY FIGURES

SupFigure 1

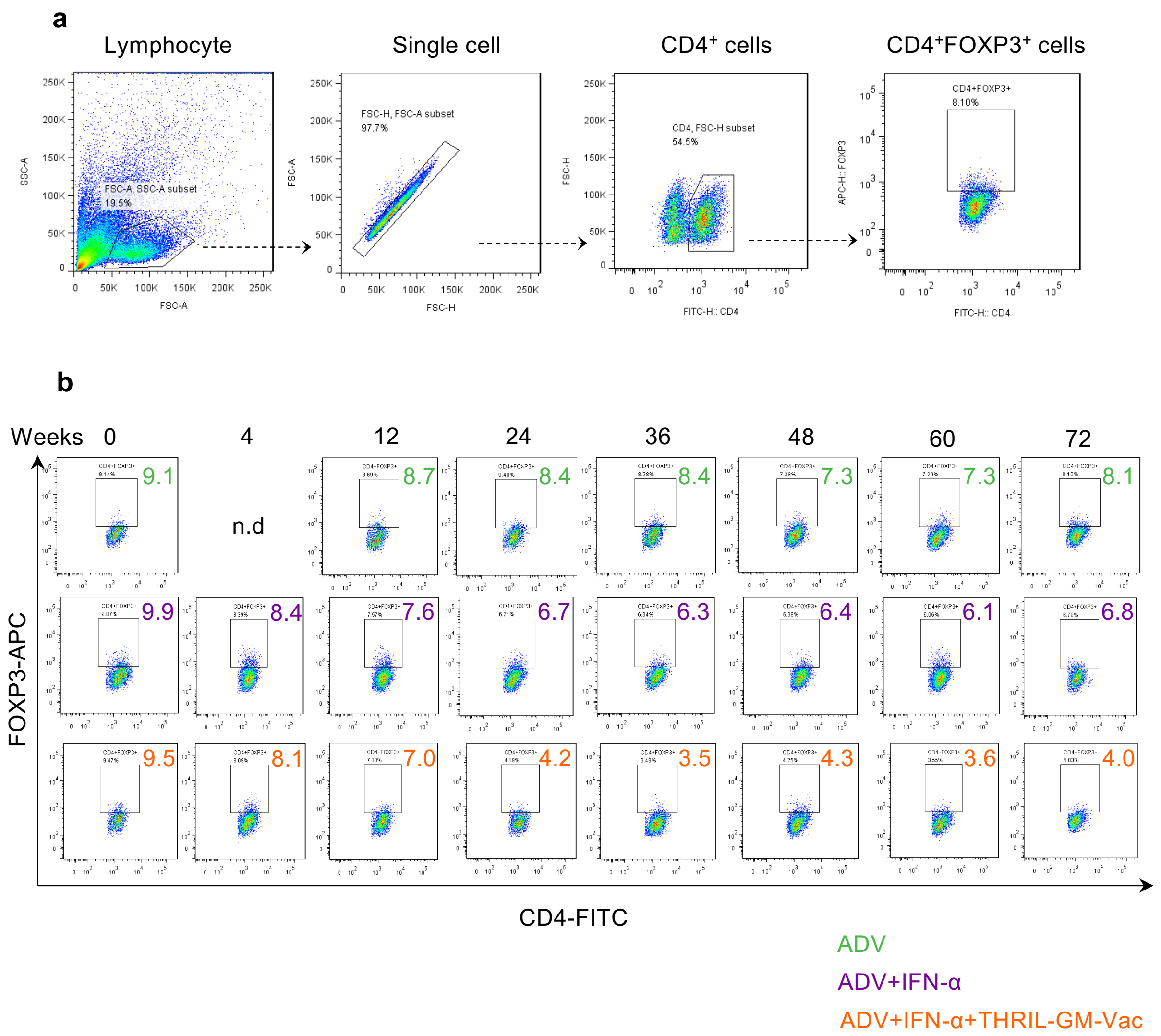

SupFigure 1. Gating Strategy for Treg Analysis and Representative Dot Plots Over the Intervention Course (Weeks 0 to 72)

(a) Gating Strategy

Flow cytometry analysis was performed on peripheral blood mononuclear cells (PBMCs) using a BD LSRFortessa flow cytometer (BD Biosciences). Lymphocytes were gated based on forward and side scatter characteristics, followed by singlet discrimination

using FSC-A vs. FSC-H plots. CD4+ T cells were identified using anti-CD4 (clone OKT4, BioLegend) staining, and regulatory T cells (Tregs) were defined as CD4+FOXP3+ cells using anti-FOXP3 (clone PCH101, eBioscience) staining. Intracellular staining for FOXP3 was performed after fixation and permeabilization with 4% paraformaldehyde (PFA) and 0.2% Triton X-100.

(b) Representative Dot Plots

Treg dynamics for one patient from each group are shown in a time series (weeks 0, 4, 12, 24, 36, 48, 60, 72). Data are representative of three independent experiments.

SupFigure 2

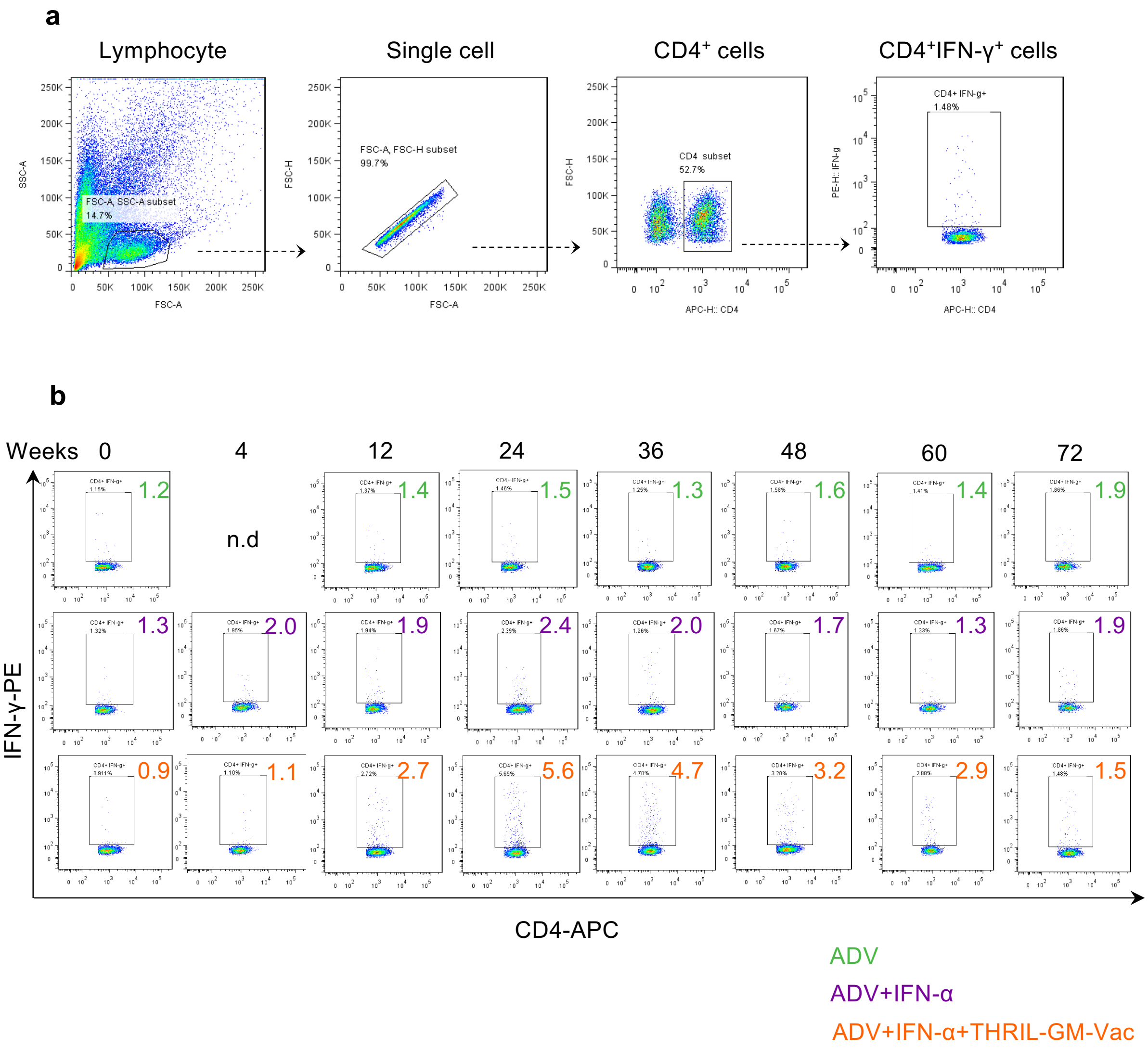

(b)

SupFigure 2. Gating Strategy for CD4+ Effector T Cell Analysis and Representative Dot Plots Over the Intervention Course (Weeks 0 to 72).

(a) Gating Strategy

PBMCs were stimulated with HBsAg peptide pools (10 μg/mL) or medium alone for 8 hours in the presence of anti-CD28 (0.1 μg/mL, Miltenyi Biotec) and brefeldin A (BD Biosciences). After surface staining with anti-CD4 (clone OKT4, BioLegend), cells were fixed, permeabilized, and stained with anti-IFN-γ (clone 4S.B3, eBioscience). CD4+ effector T cells (Teff) were defined as

CD4+IFN-γ+ cells.

(b) Representative Dot Plots

Teff dynamics for one patient from each group are shown in a time series (weeks 0, 4, 12, 24, 36, 48, 60, 72). Data are representative of three independent experiments.

SupFigure 3

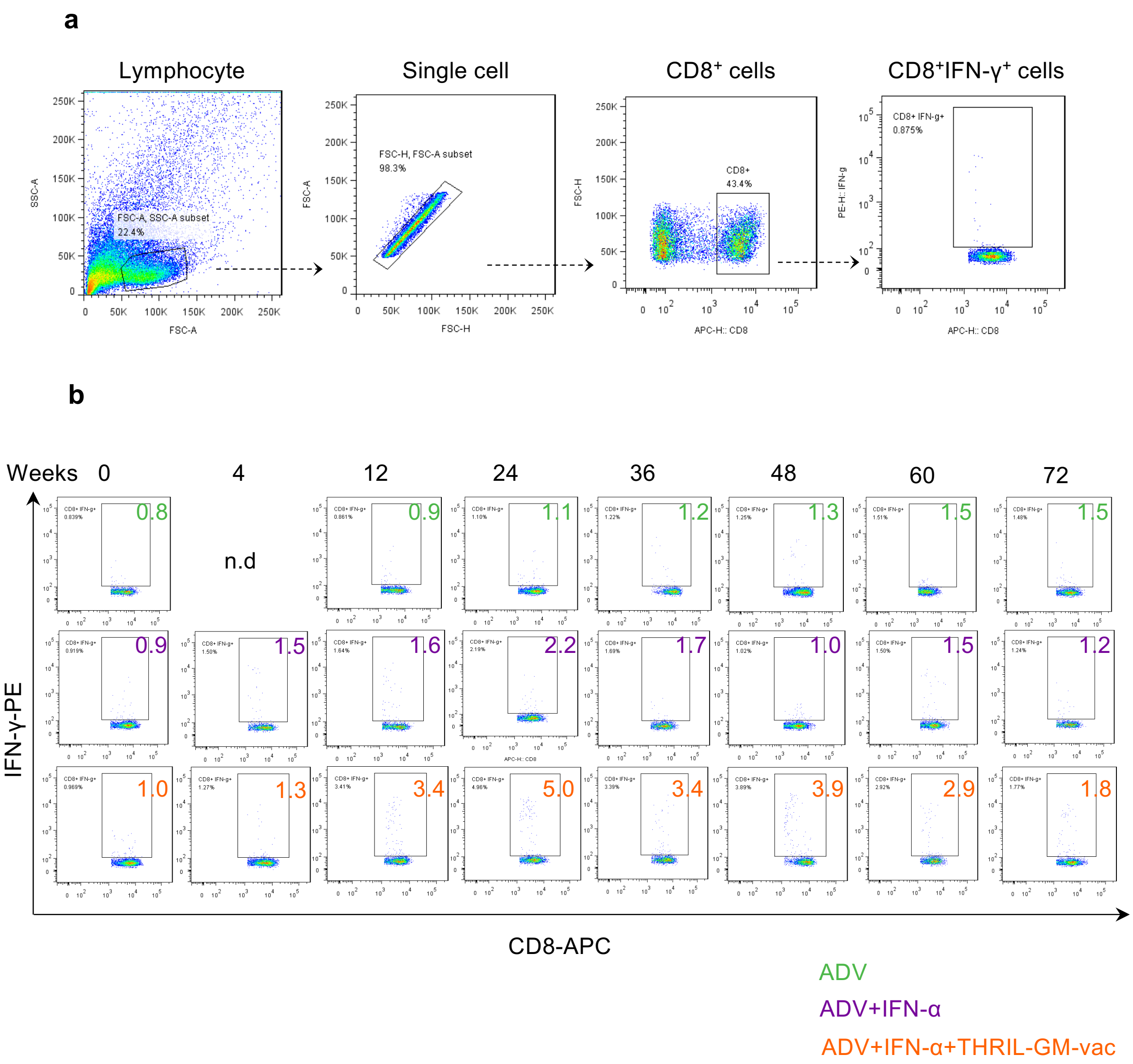

SupFigure 3. Gating Strategy for CD8+ Effector T Cell Analysis and Representative Dot Plots Over the Intervention Course (Weeks 0 to 72)

(a) Gating Strategy

PBMCs were stimulated as described in SupFigure 2. After surface staining with anti-CD8 (clone SK1, BD Biosciences), cells were fixed, permeabilized, and stained with anti-IFN-γ (clone 4S.B3, eBioscience). CD8+ effector T cells (Teff) were defined as CD8+IFN-γ+ cells.

(b) Representative Dot Plots

Teff dynamics for one patient from each group are shown in a time series (weeks 0, 4, 12, 24, 36, 48, 60, 72). Data are representative of three independent experiments.

SUPPLEMENTARY TABLE

Supplementary Table 1. HBsAg Specific Peptide Pools for T Cell In Vitro Stimulation.

Stimulating peptide subset	sequence	protein	position	HLA restriction
CD4T/Treg	FFLLTRILTI	HBsAg	19-28	DPw4/DR7
	FFLLTRILTIPQSLD	HBsAg	19-33	DR2w 15
	TSLNFLGGTTVCLGQ	HBsAg	37-51	DR1
	QSPTSNHSPTSCPPIC	HBsAg	54-69	
	CTTPAQGNSMFPSC	HBsAg	124-137	
	CTKPTDGN	HBsAg	139-146	DR1/3/4/5/6/7/11
	WASVRFSW	HBsAg	165-172	DR11/14
	LLPIFFCLW	HBsAg	215-223	DR7/8/14
$CD8^+$ T cell	VLQAGFFLL	HBsAg	14-22	A2
	FLGGTPVCL	HBsAg	41-49	A2
	LLCLIFLLV	HBsAg	88-96	A2
	LVLLDYQGML	HBsAg	95-104	A2
	LLDYQGMLPV	HBsAg	97-106	A2
	SIVSPFIPLL	HBsAg	207-216	A2
	ILSPFLPLL	HBsAg	208-216	A2

Notes:

- Peptides were synthesized by GenScript (Piscataway, NJ) and pooled based on predicted binding affinity to HLA-A*02:01 alleles using the Immune Epitope Database (IEDB) tool (https://www.iedb.org/).
- Peptide pools were used at a final concentration of 10 μg/mL for in vitro stimulation of PBMCs.
- Binding affinity was predicted using the IEDB MHC class I binding assay tool, with IC50 values <50 nM considered high-affinity binders.
